# Chinese Patent Medicine Tongxinluo Capsule for Hypertension: A Systematic Review of Randomised Controlled Trials

**DOI:** 10.1155/2014/187979

**Published:** 2014-02-17

**Authors:** Jie Wang, Xingjiang Xiong, Wei Liu

**Affiliations:** Department of Cardiology, Guang'anmen Hospital, China Academy of Chinese Medical Sciences, Beixiange No. 5, Xicheng District, Beijing 100053, China

## Abstract

*Background*. This study was intended to evaluate the efficacy and safety of Tongxinluo capsule for hypertension. *Search Strategy*. We searched the Cochrane Central Register of Controlled Trials (CENTRAL) in the Cochrane Library, The PubMed, EMBASE, Chinese Bio-Medical Literature Database (CBM), Chinese National Knowledge Infrastructure (CNKI), Chinese Scientific Journal Database, and Wan-fang Data started from the first of database to October 28, 2013. No language restriction was applied. We included randomized clinical trials testing Tongxinluo capsule against western medicine, Tongxinluo capsule versus placebo, and Tongxinluo capsule combined with western medicine versus western medicine. Study selection, data extraction, quality assessment, and data analyses were conducted according to the Cochrane standards. *Results*. 25 trials with 1958 participants were included. The methodological quality of the included trials was evaluated as generally low. The blood pressure (BP) lowering effect of Tongxinluo capsule plus western medicine was significantly higher than that of western medicine (systolic blood pressure (SBP): −3.87, −5.32 to −2.41, *P* < 0.00001; and diastolic blood pressure (DBP): −2.72, −4.19 to −1.24, *P* = 0.0003). The BP also decreased significantly from baseline with Tongxinluo capsule than placebo (SBP: −9.40, −10.90 to −7.90, *P* < 0.00001; and DBP: −11.80, −12.40 to −11.20, *P* < 0.00001) or western medicine (SBP: −3.90, −4.93 to −2.87, *P* < 0.00001; and DBP: −3.70, −3.83 to −3.57, *P* < 0.00001). 12 trials reported adverse events without details. *Conclusions*. There is some but weak evidence about the effectiveness of TXL in treating patients with hypertension.

## 1. Introduction

With an ageing worldwide population coupled with unhealthy lifestyles and increased medical intervention, the burden of disease and overall mortality has shifted gradually to primarily noncommunicable diseases such as cardiovascular diseases. Hypertension is the most common risk factor for cardiovascular diseases (CVD) [1]. It has become a growing public health concern, particularly in developing countries, with an estimated prevalence of 37.3%, in comparison with 22.9% in industrialized nations. Projections are that by the year of 2025, 75.0% (or 1.17 billion people) of the people with hypertension in the world will be living in emerging nations [2–4]. Unfortunately, the prevalence of hypertension is growing rapidly in developing countries which are undergoing epidemiological transitions, economic improvement, urbanization, and longer life expectancy [5, 6]. The prevention and management of hypertension are major public-health challenges. It is well known that lowering blood pressure decreases the risk of cardiovascular diseases (CVDs) and cerebrovascular diseases in people with moderate to severe hypertension [7–9]. In recent decades, different classes of antihypertensive agents were developed and tested in a variety of settings and among different patients. However, approximately one-half of the patients with high blood pressure (BP) are not compliant with classifications of antihypertensive agents for various reasons including treatment cost, adverse effects, and complications [10–13]. Therefore, seeking for a new effective decompression method is an important subject of hypertension treatment.

Complementary and alternative (CAM) therapies are becoming increasingly popular among patients. Complementary and alternative medicine describes the field of inquiry into therapies that are not widely taught in medical schools or generally available in hospitals [14–20]. Recent less widely prescribed but increasingly popular among patients are CAM antihypertensive therapies [21–23]. Traditional Chinese medicine (TCM) is a main component of CAM. Evidence from systematic reviews supports the blood pressure-lowering effects of TCM including herbal medicine [24], acupuncture [25, 26], moxibustion [27], Taichi [28], and Qigong [29, 30]. Tongxinluo (TXL), in capsule form, is a traditional Chinese medicine which consists of herbs and insects (*Panax ginseng, leech, scorpion, Radix paeoniae rubra, periostracum cicadae, Ground Beetle, Centipede, Sandalwood, Rosewood Heart Wood, Frankincense, Semen Ziziphi Spinosae, and Borneol*). Traditionally it has been thought to have clinical benefits for patients with angina, including the reduction of the occurrence of acute myocardial infarction (AMI), and complications of some types of heart surgery [31–35]. Some studies also suggest a possible benefit in reducing the BP and improving symptoms [36]. It has been shown to exert a variety of pharmacological effects to lower BP, including inhibiting the platelet activation [37] and vascular inflammation response, also reducing the concentration of AngII in the plasma [38] and improving the vascular endothelial function [39]. Although TXL has been commonly used in clinical practice in China for hypertension for years, no systematic review has been performed to evaluate the effectiveness of TXL for the condition. Thus, the aim of the current systematic review is to assess the effectiveness and safety of TXL for hypertension.

## 2. Materials and Methods

### 2.1. Database and Search Strategies

Literature searches were conducted in the Cochrane Central Register of Controlled Trials (CENTRAL) in the Cochrane Library (October, 2013), The PubMed, EMBASE, Chinese Bio-Medical Literature Database (CBM), Chinese National Knowledge Infrastructure (CNKI), Chinese Scientific Journal Database, and Wan-fang Data. Databases in Chinese were searched to retrieve the maximum possible number of trials of TXL for hypertension, because TXL is mainly used and researched in China. All of those searches ended on October 28, 2013. Ongoing registered clinical trials were searched in the website of international clinical trial registry by U.S. national institutes of health (http://clinicaltrials.gov/). The following search terms were used individually or combined: “hypertension,” “blood pressure,” “Tongxinluo,” “Tongxinluo capsule,” “clinical trial,” and “randomized controlled trial.” The bibliographies of included studies were searched for additional references.

### 2.2. Inclusion Criteria

All the parallel randomized controlled trials (RCTs) of all the prescriptions based on “TXL” compared with western medicine or placebo in patients with hypertension were included. RCTs of all the prescriptions based on TXL combined with western medicine compared to western medicine. Studies were excluded if they were nonrandomized studies and/or involving other forms of TXL such as TXL combined with other decoctions. There were no restrictions on population characteristics, language, and publication type. The primary outcome measures were blood pressure (BP), and the secondary outcome measure was adverse events. Duplicated publications reporting the same groups of participants were excluded.

### 2.3. Data Extraction and Quality Assessment

Two authors conducted the literature searching (W. Liu and X. J. Xiong), study selection (W. Liu and X. J. Xiong), data extraction (W. Liu and X. J. Xiong), and evaluated data's quality independently (W. Liu and X. J. Xiong). We classified trials and abstracts according to patient characteristics, study design, and therapy duration. Reviewing study design included the following criteria: methods of sequence generation, allocation concealment, complete description of those who were blinded, and use of intention-to-treat analysis and whether the trial was stopped prior to the planned duration, all methodological features in addition capable of impacting effect sizes. The extracted data included authors and title of study, year of publication, study size, age and sex of the participants, details of methodological information, treatment process, details of control interventions, outcomes (BP), and adverse events. The data was entered into an electronic database by the two reviewers separately, avoiding duplicate entries; in the case where the two entries did not match, an inspection will be conducted, and a third person may be involved for verification. In order to obtain full information regarding conference abstracts, we had contacted the study authors by email and/or telephone communication. Disagreement was resolved by discussion and reached consensus through a third party (J. Wang).

The methodological quality of trials was assessed independently using criteria from the Cochrane Handbook for Systematic Review of Interventions, Version 5.1.0 (W. Liu and X. J. Xiong) [40]. The items included random sequence generation (selection bias), allocation concealment (selection bias), blinding of participants and personnel (performance bias), blinding of outcome assessment (detection bias), incomplete outcome data (attrition bias), selective reporting (reporting bias), and other biases. The quality of all the included trials was categorized to low/unclear/high risk of bias (“Yes” for a low of bias, “No” for a high risk of bias, and “Unclear” otherwise). Then trials were categorized into three levels: low risk of bias (all the items were in low risk of bias), high risk of bias (at least one item was in high risk of bias), and unclear risk of bias (at least one item was in unclear and risk of bias).

### 2.4. Data Synthesis

Data were summarized using relative risk (RR) with 95% confidence intervals (CI) for binary outcomes or mean difference (MD) with 95% CI for continuous outcomes. We used Revman 5.1 software provided by the Cochrane Collaboration for data analyses. Studies were stratified by the type of comparison. Continuous outcome will be presented as mean difference (MD) and its 95% CI. Heterogeneity was recognized significant when *I* ^2^ ≥ 50%. Fixed effects model was used if there is no significant heterogeneity of the data; random effects model was used if significant heterogeneity existed (50% < *I* ^2^ < 85%). Publication bias was explored using a funnel plot.

## 3. Results

### 3.1. Description of Included Trials

**Figure 1:**
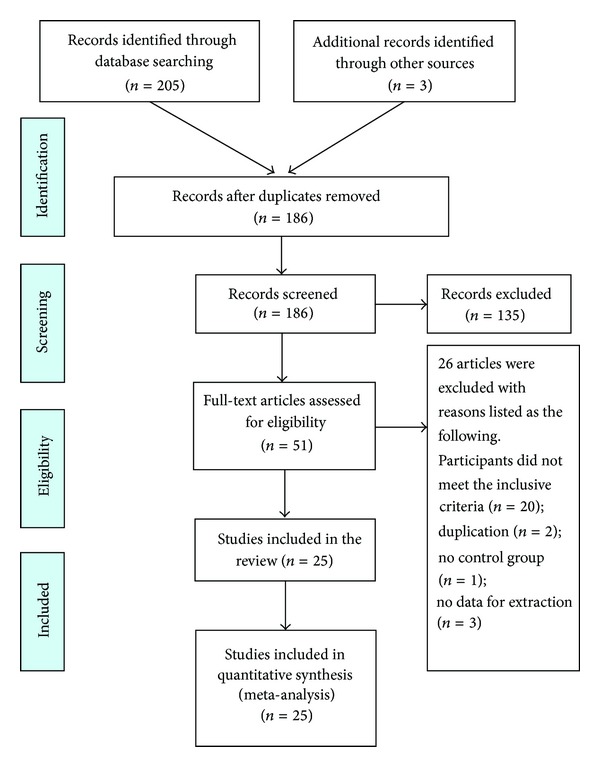
PRISMA 2009 flow diagram.

A flow chart depicted the search process and study selection (as shown in Figure 1). After primary searches from the databases, 186 articles were screened. After reading the titles and abstracts, 135 articles of them were excluded. Full texts of 25 articles [41–65] were retrieved, and 26 articles were excluded with reasons listed as the following: participants did not meet the inclusive criteria (*n* = 20), duplication (*n* = 2), no control group (*n* = 1), and no data for extraction (*n* = 3). In the end, 25 RCTs were included, and all trials had been conducted and published in China.

1958 patients with hypertension were included, with the average number of 78 per trial, ranging from 52 to 240. The proportion of male participants was 75.7%. There was a wide variation in the age of subjects (39–75 years). All trials enrolled patients with hypertension. 25 trials specified 5 diagnostic criteria of hypertension, 1 trial [50] used Chinese Guidelines for the Management of Hypertension-2000 (CGMH-2000), 2 trials [45, 46] used Chinese Guidelines for the Management of Hypertension-2004 (CGMH-2004), 6 trials [49, 51, 54, 56, 58, 64] used Chinese Guidelines for the Management of Hypertension-2005 (CGMH-2005), 12 trials [41–43, 47, 48, 52, 55, 57, 59–62] used 1999 WHO-ISH guidelines for the management of hypertension (1999 WHO-ISH GMH), 1 trial [53] used The management of arterial hypertension of the European Society of Hypertension (ESH) and the European Society of Cardiology (ESC) 2007, and 3 trials [44, 63, 65] only demonstrated patients with essential hypertension. Among the included clinical trials, 1 trial [64] compared TXL with placebo, 1 trial [65] compared TXL alone with conventional western medicine, and 23 trials [41–63] compared the combination of TXL and conventional western medicine with conventional western medicine. The total treatment duration ranged from 4 to 52 weeks. All of the 25 trials used BP as the main outcome measure. The characteristics of 25 trials were summarized in Table 1. 12 trials reported adverse events [41, 42, 47, 50–52, 55, 56, 59, 61, 63, 64].

### 3.2. Methodological Quality of Included Trials

**Figure 2:**
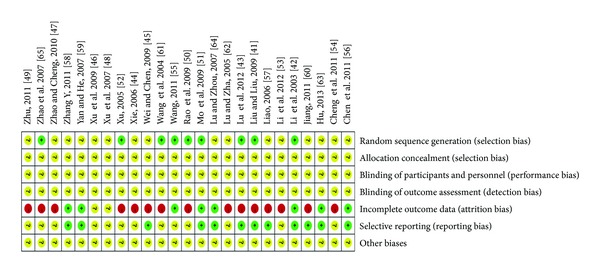
Risk of bias summary: review authors' judgements about each risk of bias item for each included study.

**Figure 3:**
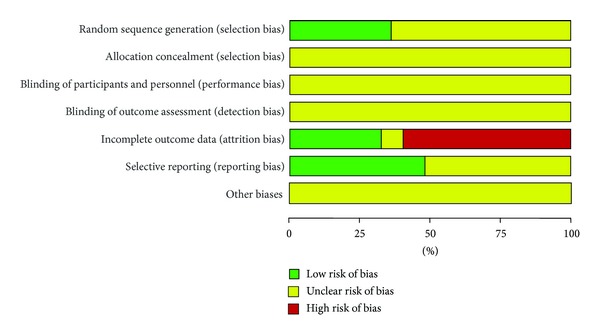
Risk of bias graph: review authors' judgements about each risk of bias item presented as percentages across all included studies.

The methodological quality of most included trials was generally “poor”. The details are as shown in Figures 2-3. All the included trials have mentioned the randomized allocation of participants; however, only 9 trials reported the methods for sequence generation including random number table [42, 43, 50, 51] and drawing [41, 52, 55, 61, 65]. No specific information was provided in the other 16 trials to judge whether or not it was conducted properly. Allocation concealment, blinding of participants and personnel, and blinding of outcome assessment were not mentioned in these 25 trials. No trials have reported dropout and a pretrial estimation of sample size. Two trials reported information on follow-up [60, 63]. We tried to contact with the authors who conducted the trials by telephone, fax, and email for further detailed information mentioned above; however, no information has been provided to date.

### 3.3. Details of Included Trials

**Figure 4:**
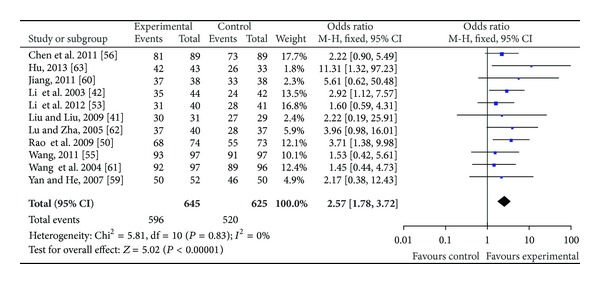
Analyses of blood pressure.

**Figure 5:**
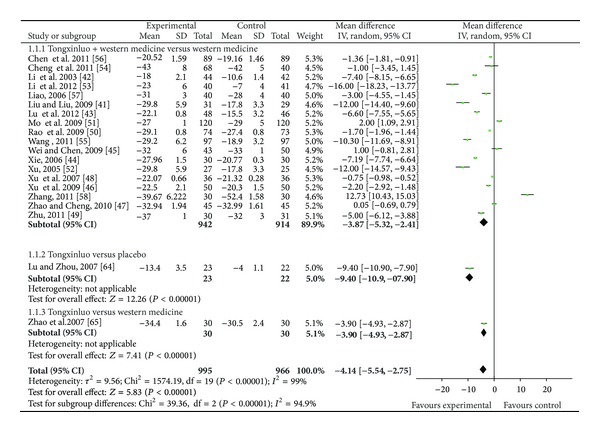
The forest plot of outcome measure SBP.

**Figure 6:**
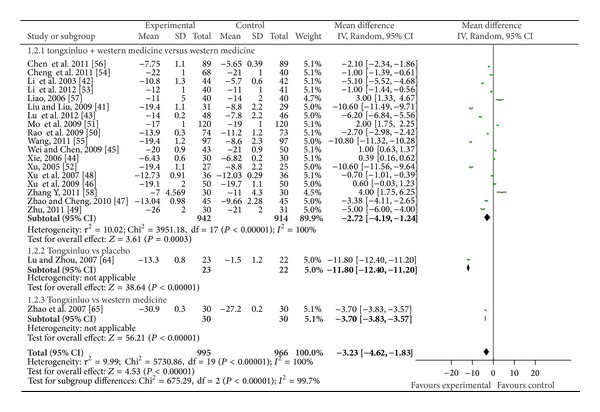
The forest plot of outcome measure DBP.

25 RCTs were included in the group of studies of patients with essential hypertension [41–65]. All the trials claimed positive effect favoring TXL though some of the trials turned out to be negative when analyzed by standard statistical techniques using risk ratios or mean differences. The effect estimates of TXL were shown in Figures 4–6.

#### 3.3.1. TXL Combined with Western Medicine versus Western Medicine

A total of 23 trials [41–63] reported the effect of TXL plus western medicine versus western medicine on blood pressure (BP). The trials showed significant difference between treatment and control group on the criteria outcome measurement (OR: 2.57 (1.78, 3.72), *P* < 0.00001) (as shown in Figure 4). When it comes to systolic blood pressure (SBP), 13 independent trials did show better effect: 6 studies [41–43, 49, 52, 55] combined with calcium channel blocker (CCB) had lower SBP than CCB alone (*P* < 0.00001). Two studies [53, 56] combined with Angiotensin Receptor Blocker (ARB) had lower SBP than ARB alone (*P* < 0.00001). One [53] significantly reduced the SBP compared to Candesartan (−16.00 mm Hg, 95% CI −18.23 to −13.77, *P* < 0.00001). The other [56] had better effect compared to valsartan capsules (−1.36, −1.81 to −0.91). Xie [44] tested the effect of TXL and Benazepril Hydrochloride Tablets (ACEI) on mild to moderate essential hypertension. The study showed a significant fall of mean blood pressure after 6 months (−7.19, −7.74 to −6.64). Four studies [46, 48, 50, 57] discovered that TXL combined with western medicine showed better effect compared to western medicine groups alone. Meta-analysis showed beneficial effect on the combination group as compared to conventional western medicine group (WMD: −3.87 [−5.32, −2.41]; *P* < 0.00001) (as shown in Figure 5).

When it comes to DBP, 12 trials demonstrated better effect favoring TXL [41–43, 47–50, 52–56]: TXL plus ARB mildly lowered DBP than ARB alone [47, 53, 54, 56]. TXL plus CCB significantly lowered DBP than CCB alone [41–43, 49, 52, 55]. Two studies [48, 50] combined with conventional western medicine had mildly lower DBP than conventional western medicine alone. One [50] mildly lowered DBP than Nifedipine controlled release tablets combined with Captopril (−2.70, −2.98 to −2.42), the other [48] decreased blood pressure compared with the control group (−0.70, −1.01 to −0.39). Meta-analysis showed no beneficial effect on the combination group as compared to conventional western medicine group (WMD: −2.72 [−4.19, − 1.24]; *P* = 0.0003) (as shown in Figure 6).

#### 3.3.2. TXL versus Placebo

Only one trial reported the effect of TXL compared with placebo in patients with essential hypertension. Lu and Zhou [64] discovered that TXL only significantly reduced both SBP and DBP (SBP: −9.40, −10.90 to −7.90, *P* < 0.00001 and DBP: −11.80, −12.40 to −11.20, *P* < 0.00001).

#### 3.3.3. TXL versus Western Medicine

Only one trial [65] showed TXL individually versus western medicine. There were statistically significant differences on the TXL group to western medicine alone (SBP: −3.90, −4.93 to −2.87, *P* < 0.00001; and DBP: −3.70, −3.83 to −3.57, *P* < 0.00001).

#### 3.3.4. Adverse Effect

The safety problem about medical measurement is getting increasing concern all over the world. As shown in Table 1, the adverse events were reported in 12 trials [41, 42, 47, 50–52, 55, 56, 59, 61, 63, 64]. 12 trials reported several specific symptoms including nausea, vomiting, stomach pains, bloating, hypotension, facial blushing, menorrhagia, and dry cough. In our review, only one trial [64] described the adverse events even without detailed information in the treatment group, and one trial [50] exported that none of the participants has any adverse events. 10 trials [41, 42, 47, 51, 52, 55, 56, 59, 61, 63] reported adverse effect in TXL group including nausea, vomiting, stomach pains, bloating, hypotension, facial blushing, and menorrhagia. Seven trials [41, 47, 52, 55, 59, 61, 63] mentioned adverse effect in control group including nausea, vomiting, stomach pains, bloating, hypotension, and dry cough. All adverse effects are not serious, and symptoms relieved and disappeared when symptomatic treatment or the drug usage is stopped or reduced.

#### 3.3.5. Publication Bias

**Figure 7:**
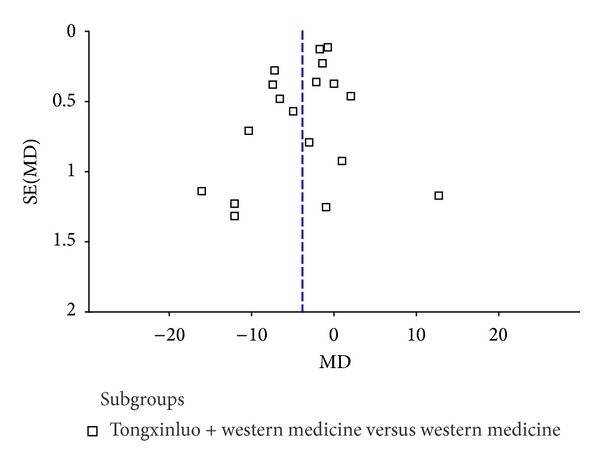
The forest plot of comparison of SBP in Tongxinluo capsule combined with western medicine versus western medicine.

**Figure 8:**
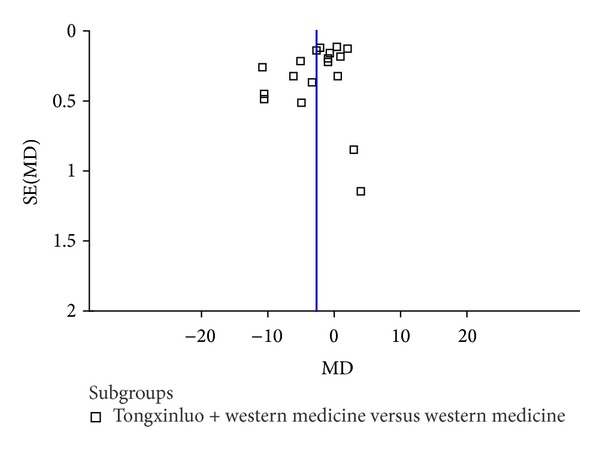
The forest plot of comparison of DBP in Tongxinluo capsule combined with western medicine versus western medicine.

The forest plot of comparison of TXL combined with western medicine versus western medicine for the outcome blood pressure was shown in Figures 7-8.

## 4. Discussion

TXL is a medicine consisting of traditional Chinese herbs and insects used for cardiovascular diseases in China and some other Asian countries. Currently, TXL used alone or combined with conventional western medicine has been widely used as an alternative and effective method for the treatment of hypertension in clinical treatment. Until now, a number of clinical trials of TXL for hypertension have been conducted and reported with positive findings [66, 67].However, there have been no systematic English literature reviews to examine this modality. To the best of our knowledge, this is the first systematic review and meta-analysis of RCTs for TXL in treating essential hypertension.

Based on the paper and meta-analyses of the outcome on either SBP or DBP, TXL may have positive effects for lowing BP. Three subgroups were analyzed based on methodological variables of TXL arms and control arms. The BP lowering effect of TXL plus western medicine was significantly higher than that of western medicine (SBP: −3.87, −5.32 to −2.41, *P* < 0.00001 and DBP: −2.72, −4.19 to −1.24, *P* = 0.0003). The BP also decreased significantly from baseline with TXL than placebo (SBP: −9.40, −10.90 to −7.90, *P* < 0.00001 and DBP: −11.80, −12.40 to −11.20, *P* < 0.00001). TXL achieved significant effect modification on BP change magnitude compared with western medicine (SBP: −3.90,-4.93 to −2.87, *P* < 0.00001 and DBP: −3.70, −3.83 to −3.57, *P* < 0.00001). However, according to potential publication bias and low-quality trials, available data are not adequate to draw a definite conclusion of TXL for essential hypertension. And the positive findings should be interpreted conservatively.

However, the following limitations should be considered before accepting the findings of this paper. As with any meta-analysis, the results were impacted by the quality of the included studies. As the insufficient information reported from the included studies, the quality of methodology is generally low in most of the included trials. Firstly, the majority of the included trials were assessed to be of general poor methodological quality according to the predefined quality assessment criteria by using criteria from the Cochrane Handbook for Systematic Review of Interventions, Version 5.1.0. The 25 trials included in this paper had risk of bias in terms of design, reporting, and methodology. Only 9 RCTs stated randomization procedure or drawing, for the rest 16 trials, they just mentioned that “the patients were randomized into two groups” with no further information. Several trials [41, 52, 55, 61, 65] used an inadequate method for sequence generation. Because inadequate sequence generation in randomisation studies also tends to yield a larger estimate of treatment effects, this is another source of potential bias. No trials have conducted allocation concealment. A number of trials [44, 49, 52, 55, 57, 60, 63] only have one author, which is impossible for an RCT to be done properly in terms of randomization procedure and the allocation concealment. Therefore, we could suspect the truth of some of these claimed RCTs. In addition, all the trials did not describe the blinding in detail. It directly led to performance bias and detection bias due to patients and researchers being aware of the therapeutic interventions for the subjective outcome measures. None of them has reported dropout or withdraw. This will undermine the authenticity and reliability of the studies. As none of them had a pretrial estimation of sample size, whether the sample meets the requirements is still unclear. Hypertension is a chronic disease which is a great concern of patients about the effect of long-term treatment. Indeed, only 2 trials had mentioned the follow-up without detailed information. Moreover, all the included 11 trials were not multicenter, large-scale RCTs which may have resulted in performance bias. Although we had tried to search more detailed information, no information could be got. These factors may have a potential impact on our results. If poorly designed, all the trials would show larger differences compared with well designed trials [68, 69].

Secondly, the heterogeneity in the included trials may have influenced the conclusions. Many factors affect the effects of heterogeneity, such as dosages of TXL, frequency and duration of the treatment sessions, the kinds of western medicine, and the course of hypertension. One of the major limitations was the application of various kinds of dosages. Five trials [43, 44, 59, 61, 65] used TXL (0.38 g/pill), and the dosage (0.26 g/pill) was applied in two trials [50, 51]. Others did not describe the dosages of treatment group. In addition, the differences of frequency and duration of the treatment sessions (ranging from 2 pills to 4 pills and 4 to 52 weeks) affected the effects of TXL. Among them, 13 trials [41, 42, 47–49, 51–54, 57, 58, 60, 64] used TXL 3 pills each time, three times a day; the frequency and duration (4 pills each time, three times a day) were applied in six trials [43–45, 50, 56, 62], four trials [46, 55, 59, 61] described TXLs were used by 3 pills each time, twice a day, and only one trial [65] used TXL by 4 pills each time, twice a day. Thus, they made contributions to the great0 heterogeneity. Furthermore, the difference of kinds of western medicine is another important factor which caused heterogeneity. Medical interventions for those with severe hypertension generally have to use antihypertensive drugs such as diuretics, *α*- and *β*-blockers, angiotensin-converting enzyme inhibitors, and long-acting calcium-channel blockers [70]. CCB were applied to seven trials [41–43, 49, 52, 55, 59] combined with TXL in the treatment group, five trials [47, 53, 54, 56, 58] used ARB, and ACEI of western medicine were applied in five trials [44, 51, 60, 63, 65]. Others did not account detailed information [45, 46, 48, 57, 62] of using conventional western medicine. Not only that, all trails specified 5 diagnostic criteria of hypertension without 3 and there were different courses of hypertension (ranged from 1 to 23); selective reporting bias might exist in this conclusion and reduce the homogeneity of the research objects. The all the 25 RCTs prohibited us to perform meaningful sensitivity analysis.

Moreover, publication bias cannot be fully excluded because, without sufficient studies, the Begg and Egger tests have low power to detect publication bias [71–74]. Funnel plot (Figures 7-8) indicated that publication bias would exist in this review. It is conceivable that several negative RCTs remain unpublished, thus distorting the overall picture [71]. The reasons we considered were as follows: we searched the Cochrane Library, PubMed, EMBASE, CBM, CNKI, Chinese Scientific Journal Database, and Wan-fang Data until October 28, 2013. In addition, the online clinical trial registry websites were also searched. However, all included studies were conducted in China. Most of the studies are small size with positive findings. We only selected trials published in Chinese and trials published in other languages or originated from other countries might be omitted; we only identified unpublished studies from conference paper or academic thesis, and negative trials might not be reported and induce publication bias. In addition, to evaluate the efficacy of clinical trials needs follow-up for a long-term, according to the characteristic of the chronic diseases. However, only 2 trials [60, 63] included in the review had followed up for 3 months or 1 year without results and valid data. Therefore, in order to properly assess the long-term outcomes of TXL for hypertension, clinical trials with more rigorous designs are required.

As we know, the safety problem is an important guarantee for treatment measures [75, 76]. The safety of Chinese herbal medicines is of general concern. As a result, a conclusion about the safety of TXL could not be made. 13 trials of all [41–44, 47] did not mention whether they had monitored adverse effects at all and one trial [50] mentioned the two groups did not appear adverse events. Five trials [41, 52, 55, 56, 64] did not discover liver and renal toxicity. The adverse effects were reported in both intervention group and control group, including nausea, vomiting, stomach pains, bloating, hypotension, and dry cough, which might be related to the adverse effect of western medicine. Four trials [41, 52, 55, 59] reported facial blushing in the Chinese herbs group, and three cases of menorrhagia were identified in one trial [61].

## 5. Conclusions

There is some but weak evidence about the effectiveness of TXL in treating patients with hypertension. However, the total number of RCTs included in the analysis, the total sample size, and their risk of bias were quite high in several domains; thus, drawing firm conclusions concerning the effectiveness of TXL therapies remains difficult. Further rigorous RCTs that should follow the Consolidated Standards of Reporting Trials (CONSORT) Statement [77] are warranted.

## Figures and Tables

**Table 1:** Characteristics and methodological quality of included studies.

Study ID	Sample	Age	Diagnosis standard	Intervention	Control	Course	Outcome measure
	(M/F)	(yrs)				(week)	
F.M. Liu and C. F. Liu, 2009 [41]	60 T: 21/10; C: 20/9	41 to 76 (T/C not reported)	1999 WHO-ISH GMH	TXL + C	Amlodipine (5 mg qd)	8 (3 pills/time, tid)	BP; Adverse event
Li et al. 2003 [42]	86 T: 44; C: 42 M/F: 54/32	60 to 75 (T/C not reported)	1999 WHO-ISH GMH	TXL + C	Nifedipine controlled released tablets (30 mg qd)	12 (3 pills /time, tid)	BP; Adverse event
Lu et al. 2012 [43]	94 T: 48; C: 46 M/F: 59/35	57.8 ± 11. (T/C not reported)	1999 WHO-ISH GMH	TXL + C	Nifedipine controlled released tablets (30 mg qd)	24 (0.38 g/pill, 4 pills/time, tid)	BP
Xie, 2006 [44]	60 T: 18/12 C: 20/10	T: 64 ± 10.13 C: 65 ± 9.56	Hypertension diagnostic criteria (unclear)	TXL + C	Benazepril Hydrochloride tablets (10 mg qd)	24 (0.38 g/pill, 4 pills/time, tid)	BP
Wei and Chen, 2009 [45]	93 T: 18/25 C: 21/29	T: 56 ± 8 C: 56 ± 8	Chinese Guidelines for the Management of Hypertension-2004 (CGMH-2004)	TXL + C	Western medicine	24 (4 pills/time, tid)	BP
Xu et al. 2009 [46]	100 T: 36/14 C: 38/12	40–79 (T/C not reported)	Chinese Guidelines for the Management of Hypertension-2004 (CGMH-2004)	TXL + C	Western medicine	24 (3 pills/time, bid)	BP
Zhao and Cheng, 2010 [47]	90 T: 26/19 C: 33/12	T: 66 ± 3.1 C: 64 ± 3.9	1999 WHO-ISH GMH	TXL + C	Irbesartan tablets (150 mg qd)	24 (3 pills/time, tid)	BP; Adverse event
Xu et al. 2007 [48]	72 T: 36; C: 36 M/F: 59/35	57.01 ± 8.56 (T/C not reported)	1999 WHO-ISH GMH	TXL + C	Western medicine	8 (3 pills/time, tid)	BP
Zhu, 2011 [49]	61 T: 30; C: 31 (M/F not reported)	45–70 (T/C not reported)	Chinese Guidelines for the Management of Hypertension-2005 (CGMH-2005)	TXL + C	Felodipine sustained release tablets (2.5–5.0 mg)	6 (3 pills/time, tid)	BP
Rao, 2009 [50]	147 T: 41/33 C: 39/34	T: 43.36 ± 12.47 C: 42.82 ± 11.64	Chinese Guidelines for the Management of Hypertension-2000 (CGMH-2000)	TXL + C	Nifedipine controlled release tablets (10 mg bid); Captopril (25 mg tid)	12 (0.26 g/pill, 4 pills/time, tid)	BP; Adverse event
Mo et al. 2009 [51]	240 T: 79/41 C: 76/44	T: 58 ± 12 C: 58 ± 12	Chinese Guidelines for the Management of Hypertension-2005 (CGMH-2005)	TXL + C	Fosinopril sodium tablets (10 mg qd)	12 (0.26 g/pill, 3 pills/time, tid)	BP; Adverse event
Xu, 2005 [52]	52 T: 19/8 C: 18/7	T: 40–76 C: 41–76	1999 WHO-ISH GMH	TXL + C	Amlodipine (5 mg qd)	8 (3 pills/time, tid)	BP; Adverse event
Li et al. 2012 [53]	81 T: 40 C: 41 M: 81	65 to 80 (T/C not reported)	The management of arterial hypertension of the European Society of Hypertension (ESH) and the European Society of Cardiology (ESC) 2007	TXL + C	Candesartan (8 mg qd)	12 (3 pills/time, tid)	BP
Cheng et al. 2011 [54]	108 T: 39/29 C: 23/17	T: 58 ± 8 C: 58 ± 9	Chinese Guidelines for the Management of Hypertension-2005 (CGMH-2005)	TXL + C	Losartan (50–100 mg qd)	4 (3 pills/time, tid)	BP
Wang, 2011 [55]	194 T: 57/40 C: 58/39	T: 42–74 C: 41–73	1999 WHO-ISH GMH	TXL + C	Nifedipine sustained release tablets (10 mg bid)	4 (3 pills/time, bid)	BP; Adverse event
Chen et al. 2011 [56]	178 T: 49/40 C: 47/42	T: 60.03 ± 9.16 C: 61.34 ± 8.80	Chinese Guidelines for the Management of Hypertension-2005 (CGMH-2005)	TXL + C	Valsartan capsules (80–160 mg qd)	24 (4 pills/time, tid)	BP; Adverse event
Liao, 2006 [57]	80 T: 22/18 C: 24/16	T: 50.1 ± 8.4 C: 50.4 ± 9.6	1999 WHO-ISH GMH	TXL + C	Western medicine	16 (3 pills/time, tid)	BP
Zhang, 2011 [58]	60 T: 30 C: 30 (M/F not reported)	(T/C not reported)	Chinese Guidelines for the Management of Hypertension-2005 (CGMH-2005)	TXL + C	Valsartan (160 mg qd)	8 (3 pills/time, tid)	BP
Yan and He, 2007 [59]	102 T: 32/20 C: 29/21	T: 40–74 C: 41–75	1999 WHO-ISH GMH	TXL + C	Amlodipine Benzenesulfonate tablets (5 mg qd)	4 (0.38 g/pill, 3 pills/time, bid)	BP; Adverse event
Jiang, 2011 [60]	76 T: 26/12 C: 24/14	T: 45 ± 22 C: 48 ± 25	1999 WHO-ISH GMH	TXL + C	Benazepril (10 mg qd)	24 (3 pills/time, tid)	BP
Wang et al. 2004 [61]	193 T: 58/39 C: 57/39	T: 41–73 C: 40–73	1999 WHO-ISH GMH	TXL + C	Nifedipine sustained release tablets (10 mg bid)	4 (0.38 g/pill, 3 pills/time, bid)	BP; Adverse event
Lu and Zha, 2005 [62]	77 M/F: 37/40 (T/C not reported)	57.35 ± 13.32	1999 WHO-ISH GMH	TXL + C	Western medicine	8 (4 pills/time, tid)	BP
Hu, 2013 [63]	76 T: 28/15 C: 19/14	T: 39–73 C: 34–65	Hypertension diagnostic criteria (unclear)	TXL + C	Enalapril (2.5 mg bid)	52 (2–4 pills/time, tid)	BP; Adverse event
Lu and Zhou, 2007 [64]	45 T: 23 C: 22 M/F: 25/20 (T/C not reported)	61.8 ± 6.9 (T/C not reported)	Chinese Guidelines for the Management of Hypertension-2005 (CGMH-2005)	TXL	Placebo	6 (3 pills/time, tid)	BP; Adverse event
Zhao et al. 2007 [65]	60 T: 30 C: 30 M/F: 34/26 (T/C not reported)	55–75 (T/C not reported)	Hypertension diagnostic criteria (unclear)	TXL	Captopril (50 mg bid)	4 (0.38 g/pill, 4 pills/time, bid)	BP
